# Food Protective Effects of 3-Methylbenzaldehyde Derived from *Myosotis arvensis* and Its Analogues against *Tyrophagus putrescentiae*

**DOI:** 10.1038/s41598-017-07001-5

**Published:** 2017-07-26

**Authors:** Jun-Hwan Park, Na-Hyun Lee, Young-Cheol Yang, Hoi-Seon Lee

**Affiliations:** 1 Department of Bioenvironmental Chemistry, Chonbuk National University, Jeonju, 54896 Korea; 2 School of Chemical Engineering, Chonbuk National University, Jeonju, 54896 Korea

## Abstract

The potential abilities of 3-methylbenzaldehyde derived from *Myosotis arvensis* oil and its structural analogues to act as new acaricide and mite kit (mite color deformation) against *Tyrophagus putrescentiae* (Schrank) were evaluated in the present study. Based on the LD_50_ values, 2,4,5-trimethylbenzaldehyde (0.78 μg/cm^3^) had highest vapor action against *T*. *putrescentiae*, followed by 2,4-methylbenzaldehyde (1.14 μg/cm^3^), 2,5-dimethylbenzaldehyde (1.29 μg/cm^3^), 2-methylbenzaldehyde (1.32 μg/cm^3^), 2,3-dimethylbenzaldehyde (1.55 μg/cm^3^), 3-methylbenzaldehyde (1.97 μg/cm^3^), and 4-methylbenzaldehyde (2.34 μg/cm^3^). The color deformation of seven methylbenzaldehyde analogues mixed with 2,3-dihydroxybenzaldehyde against *T*. *putrescentiae* showed mite color deformation, from coloress to reddish brown, and valuable to distinguish with the naked eye. In addition, there was no antagonistic interactions between 2,3-dihydroxybenzaldehyde and the methylbenzaldehyde analogues. These finding suggests that the methylbenzaldehyde analogues could be developed as dual functional agent to protect from fall in the commercial value of stored food products.

## Introduction

*Tyrophagus putrescentiae* (Schrank), commonly known as a cosmopolitan species of stored food mites, is found infesting a wide range of foods containing a high amount of protein and fat, such as cheese, cured ham, dried eggs, and nuts^1^. In addition, *T*. *putrescentiae* is the most predominant species associated with pet foods in Australia, Europe and the United States and is considered as a factor of allergens for dogs diagnosed with atopic dermatitis^2–4^. Infestation with *T*. *putrescentiae* has been also suggested to cause a serious storage problem for dry-cured hams^5^, dried fruits^6^, and seeds^7^, because their presence limits the salability of valuable products. In spite of the importance of stored food mites in stored products, natural acaricides against *T*. *putrescentiae* have not been specifically developed and registered in the past few decades^8, 9^. Historically, the control of stored food mites has largely depended on broad-spectrum pesticides that were originally developed and registered to control stored-product insects^10, 11^. Many insecticides against stored-product insects exhibited acaricidal activity too^8–11^. Therefore, the control of stored food mites is mainly accomplished by the use of organophosphates (lindane, malathion, and pirimiphos-methyl) and pyrethroid insecticides^12^. However, some organophosphates have been banned, because of their toxicity in human^13^ and the development of resistant mite population^14–16^. In addition, stored food mites have been reported to be significantly tolerant to pyrethroids^11, 17, 18^. In this regard, developing new agents for controlling stored food mites to prevent the degradation of valuable foods/grains is significantly challenging.

Plants and their related constituents have been studied as an alternative to synthetic acaricides, antimicrobials and insecticides because of the abundant materials used as herbal medicines^20–23^. Plant essential oils, which are hydrophobic mixtures of plant metabolites, are widely used as fragrances and flavors in perfumery, aromatherapy, cosmetics, incense, herbal medicine, household cleaning agents, foods, and drinks^19–22^. Furthermore, plant oils are increasingly being utilized as natural agents against insects and mite species^20–24^. Several studies have on focused plant essential oils for controlling stored food mites as acaricides. The acaricidal activities to *Tyrophagus longior* of essential oils from *Lavandula stoechas*, *L*. *angustifolia*, *Eucalyptus globulus*, and *Mentha piperita* and components of essential oils such as eucalyptol, fenchone, linalool, linalyl acetate, menthone, and menthol were determined in laboratory tests^23^. Studies with *Pinus pinea* ^24^ and *Cnidium officinale* ^25^ oils suggest that they are promising as acaricides against *T*. *putrescentiae*. Yang *et al*.^22^ reported that the benzaldehyde analogues derived from *Morinda officinalis* have potent toxicities against *Haemaphysalis longicornis* and *Dermatophagoides* spp. *Myosotis arvensis* (Boraginaceae) is distributed in western Eurasia and New Zealand. *M*. *arvensis* oil was historically used to exert antibacterial, antidepressant, antifungal, anti-inflammatory, and anxiolytic properties^19^. Nevertheless, *M*. *arvensis* oil lack scientific evidence that specifically explains acaricidal effect and mite kit against stored food mites. We performed this study to assess the food protective effects of 3-methylbenzaldehyde derived from *M*. *arvensis* oil and its structural analogues and color deformation against *T*. *putrescentiae*. In addition, the structural relationship of the methylbenzaldehyde analogues with mite kit was evaluated on the synergistic or antagonistic interactions in terms of acaricidal effect and color deformation.

## Results and Discussion

The essential oils of *M*. *arvensis* aerial parts and seeds were extracted with a yield of 0.081 and 0.046%, respectively. The acaricidal toxicities of the essential oils of *M*. *arvensis* aerial parts and seeds were evaluated to determine the vapor and contact actions of *M*. *arvensis* oils against *T*. *putrescentiae* (Table 1). The commonly used benzyl benzoate served as positive control of comparison in toxicity tests. In comparison with the LD_50_ value for the vapor action, the essential oils of *M*. *arvensis* aerial parts (LD_50_, 7.78 μg/cm^3^) and seeds (12.72 μg/cm^3^) were about 2.02 and 1.23 times more toxic than benzyl benzoate (15.74 μg/cm^3^) as a positive control against *T*. *putrescentiae*. For the contact action, the essential oil of *M*. *arvensis* aerial parts (6.33 μg/cm^2^) and seeds (10.38 μg/cm^2^) were 1.90 and 1.16 times more active than benzyl benzoate (12.0 μg/cm^2^). The negative control, designated as acetone, exhibited no toxicity against *T*. *putrescentiae* with the vapor and contact actions.

**Table 1:** Acaricidal toxicities of *M*. *arvensis* aerial part oil, *M*. *arvensis* seed oil, and synthetic acaricide against *T*. *putrescentiae* (^a^LD_50_ is the average of 5 determinations, with 30 adult mites per replication; Exposed for 24 h).

Samples	Bioassay	LD_50_^a^	95% CL	Slope	χ^2^ value (df, *p*)	RT^b^
*M*. *arvensis* aerial part oil	Vapor (μg/cm^3^)	7.78	6.28–9.44	2.85 ± 0.37	4.646 (4, 0.326)	2.02
	Contact (μg/cm^2^)	6.33	5.17–7.55	3.11 ± 0.41	6.097 (4, 0.192)	1.90
*M*. *arvensis* seed oil	Vapor (μg/cm^3^)	12.72	9.82–14.33	3.14 ± 0.39	2.987 (4, 0.560)	1.23
	Contact (μg/cm^2^)	10.38	8.11–12.37	2.59 ± 0.35	6.607 (5, 0.158)	1.16
Benzyl benzoate	Vapor (μg/cm^3^)	15.74	13.16–18.75	3.22 ± 0.45	1.579 (4, 0.813)	1.00
	Contact (μg/cm^2^)	12.0	10.56–14.01	3.12 ± 0.44	1.688 (4, 0.793)	1.00
Negative control	Vapor (μg/cm^3^)	—	—	—	—	—
	Contact (μg/cm^2^)	—	—	—	—	—

To further explore the acaricidal activities of two types of the essential oils against *T*. *putrescentiae*, the components of the essential oils of *M*. *arvensis* aerial parts and seeds were investigated by GC-MS analysis. The components identified by GC-MS analysis, their retention time, retention index, and area percentages are displayed in Table 2. The major components in the essential oil of *M*. *arvensis* aerial parts were 3-methylbenzaldehyde (10.18%), oleamide (9.37%), dodecane (6.51%), acetoxyacetic acid, undecyl ester (6.34%), hexachloroethane (6.21%), 2-hexyl-1-octanol (5.76%), 1-tridecanol (5.74%) and 3-decen-1-ol (5.28%). In the essential oil of *M*. *arvensis* seeds, the major components were β-farnesene (16.52%), oleamide (14.12%), butyl isothiocyanate (12.20%), hexadecanoic acid (9.34%), and phenylacetaldehyde (6.97%). Previous investigations into the essential oils of *M*. *arvensis* collected in different regions of the world have found the major components to be 3-methylbenzaldehyde (42.76%), hexadecanoic acid (15.18%), 2-hexyl-1-octanol (11.89%), and 4-nitrophenyl ester *o*-toluic acid (7.47%)^19^. In this regard, some constituents of the essential oils derived from herb plants are influenced by various internal or external factors such as the geographical location, extraction method, plant species, plant parts, and harvest time as well as storage time of plants^26, 27^.

**Table 2:** Analysis of the components of the essential oils of the *Myosotis arvensis* aerial parts and seeds (^a^ *M*. *arvensis* aerial parts; ^b^ *M*. *arvensis* seed).

Compounds	Retention time (min)	Retention Index	DB-5	Peak area (%)		Molecular mass (g/mol)	Molecular formula
	A^a^	B^b^		A	B		
3-Chloro-2,4-pentanedione	4.31	—	931	3.30	—	134.56	C_5_H_7_ClO_2_
2-Methylcyclopentanol	4.62	—	949	4.89	—	100.16	C_6_H_12_O
Butyl isothiocyanate	—	4.95	975	—	12.20	115.19	C_5_H_9_NS
3-Octanone	—	5.75	988	—	4.58	128.21	C_8_H_16_O
2-Pentylfuran	6.03	6.02	998	3.65	4.11	138.21	C_9_H_14_O
1,2,3-Trimethylbenzene	—	6.11	1020	—	4.95	120.19	C_9_H_12_
Phenylacetaldehyde	7.02	7.03	1026	3.66	6.97	120.15	C_8_H_8_O
2,4-Dimethylundecane	7.57	—	1185	3.55	—	184.36	C_13_H_28_
Hexachloroethane	7.64	—	1058	6.21	—	236.72	C_2_Cl_6_
Octanal	7.71	7.90	1005	1.87−	3.73	128.21	C_8_H_16_O
Nonanal	—	8.10	1104	—	4.58	142.24	C_9_H_18_O
Diacetone alcohol	—	8.22	1351	—	2.87	116.16	C_6_H_12_O_2_
3-Methylbenzaldehyde	8.94	—	1083	10.18	—	120.15	C_8_H_8_O
Acetoxyacetic acid, undecyl ester	9.05	—	1634	6.34	—	272.38	C_15_H_28_O_4_
1,1,3,5-Tetramethylcyclohexane	9.20	—	976	4.59	—	140.15	C_10_H_20_
1-Tridecanol	9.26	—	1229	5.74	—	200.36	C_13_H_28_O
6-Methyloctahydrocoumarin	9.70	—	1388	2.96	—	168.23	C_10_H_16_O_2_
Dodecane	9.75	9.76	1214	6.51	4.89	170.34	C_12_H_26_
3-Decen-1-ol	10.75	—	1235	5.28	—	156.26	C_10_H_20_O
Pentadecane	—	11.37	1413	—	5.73	212.42	C_15_H_32_
β-Farnesene	—	13.70	1440	—	16.52	204.35	C_15_H_24_
Tetradecanoic acid	17.44	—	1769	4.58	—	228.37	C_14_H_28_O_2_
Hexadecanoic acid	19.60	19.42	1968	4.57	9.34	256.43	C_16_H_32_O_2_
2-Hexyl-1-octanol	20.12	—	2071	5.76	—	214.39	C_14_H_30_O
2-Phenyl-2-imidazoline	23.17	—	1587	4.89	—	146.19	C_9_H_10_N_2_
Oleamide	23.52	23.54	2228	9.37	14.12	281.48	C_18_H_35_NO
Total identified				96.03	94.59		

The acaricidal activities of twenty major commercial constituents (butyl isothiocyanate, 3-chloro-2,4-pentanedione, diacetone alcohol, dodecane, hexachloroethane, hexadecanoic acid, 3-methylbenzaldehyde, nonanal, octanal, 3-octanone, oleamide, 2-pentylfuran, pentadecane, phenylacetaldehyde, 2-phenyl-2-imidazoline, tetradecanoic acid, 1,1,3,5-tetramethylcyclohexane, 1-tridecanol, and 1,2,3,-trimethylbenzene) derived from the two essential oils of *M*. *arvensis* aerial parts and seeds were evaluated using vapor bioassays against *T*. *putrescentiae* (Table 3). Based on the LD_50_ values of butyl isothiocyanate, 3-methylbenzaldehyde, nonanal, and 3-octanal in two essential oils using the vapor bioassay were 2.62, 1.97, 4.96, and 3.23 μg/cm^3^ respectively, and several constituents, including 3-chloro-2,4-pentanedione, diacetone alcohol, dodecane, hexachloroethane, hexadecanoic acid, octanal, oleaminde, 2-pentylfuran, phenylacetaldehyde, 2-phenyl-2-imidazoline, pentadecane, tetradecanoic acid, 1,1,3,5-tetramethylcyclohexane, 1,2,3-trimethylbenzene, and 1-tridecanol (>19.5 μg/cm^3^), failed to show a acaricidal effect even at the highest concentrations tested.

**Table 3:** Acaricidal toxicity of hydroxybenzaldehyde analogues, methylbenzaldehyde analogues and synthetic acaricide against *T*. *putrescentiae*, using a vapor bioassay (^a^LD_50_ is the average of 5 determinations, with 30 adult mites per replication; Exposed for 24 h).

Compounds	LD_50_ (μg/cm^3^)^a^	95% CI	Slope	χ^2^ value (df, *p*)
Butyl isothiocyanate	2.62	2.02–3.42	2.21 ± 0.48	1.305 (4, 0.253)
3-Chloro-2,4-pentanedione	>19.50	—	—	—
Diacetone alcohol	>19.50	—	—	—
Dodecane	>19.50	—	—	—
Hexachloroethane	>19.50	—	—	—
Hexadecanoic acid	>19.50	—	—	—
3-Methylbenzaldehyde	1.97	1.54–2.38	2.79 ± 0.38	8.034 (6, 0.236)
Nonanal	4.96	4.15–6.39	2.44 ± 0.41	1.764 (4, 0.623)
Octanal	>19.50	—	—	—
3-Octanone	3.23	2.45–3.92	2.11 ± 0.36	3.348 (3, 0.341)
Oleamide	>19.50	—	—	—
2-Pentylfuran	>19.50	—		
Phenylacetaldehyde	>19.50	—	—	—
2-Phenyl-2-imidazoline	>19.50	—	—	—
Pentadecane	>19.50	—	—	—
Tetradecanoic acid	>19.50	—	—	—
1,1,3,5-Tetramethylcyclohexane	>19.50	—	—	—
1,2,3-Trimethylbenzene	>19.50	—	—	—
1-Tridecanol	>19.50	—	—	—
Negative control	>19.50	—	—	—

**Figure 1:**
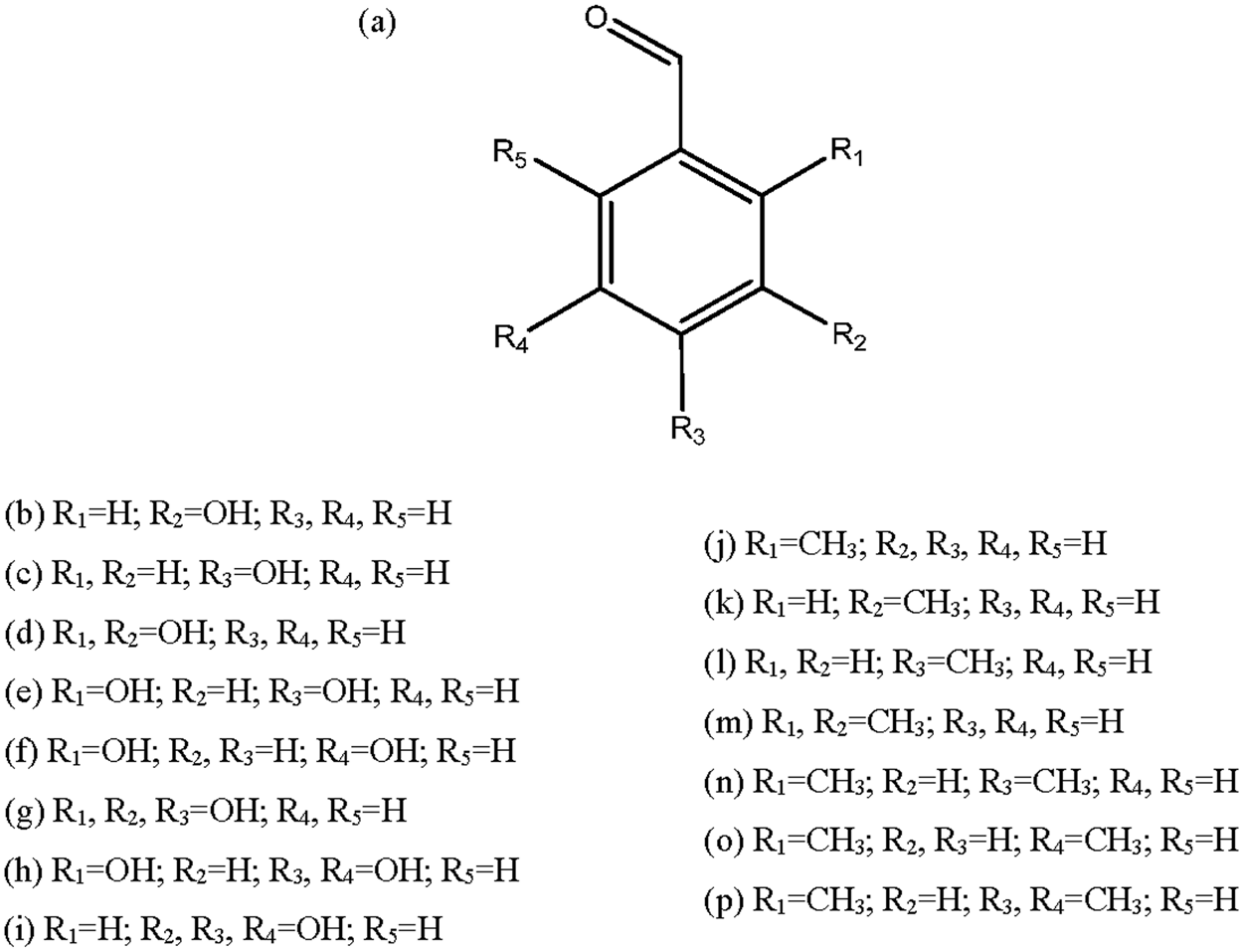
Structures of hydroxybenzaldehyde and methylbenzaldehyde analogues. (**a**) Benzaldehyde; (**b**) 3-hydroxybenzaldehyde; (**c**) 4-hydroxybenzaldehyde; (**d**) 2,3-dihydroxybenzladehyde; (**e**) 2,4-dihydroxybenzladehyde; (**f**) 2,5-dihydroxybenzladehyde; (**g**) 2,3,4-trihydroxybenzladehyde; (**h**) 2,4,5-trihydroxybenzladehyde; (**i**) 3,4,5-trihydroxybenzladehyde; (**j**) 2-methylbenzaldehyde; (**k**) 3-methylbenzaldehyde; (**l**) 4-methylbenzaldehyde; (**m**) 2,3-dimethylbenzladehyde; (**n**) 2,4-dimethylbenzladehyde; (**o**) 2,5-dimethylbenzladehyde; (**p**) 2,4,5-trimethylbenzaldehyde.

Due to the potent toxicity of 3-methylbenzaldehyde derived from essential oil of *M*. *arvensis* aerial part, the structure-toxicity relationships between the methylbenzaldehyde/hydroxybenzaldehyde analogues and acaricidal toxicities against *T*. *putrescentiae* were pursued. 3-Hydroxybenzaldehyde, 4-hydroxybenzaldehyde, 2,3-hydroxybenzaldehyde, 2,4-dihydroxybenzaldehyde, 2,5-dihydroxybenzaldehyde, 2,3,4-trihydroxybenzaldehyde, 2,4,5-trihydroxybenzaldehyde, 3,4,5-trihydroxybenzaldehyde, 2-methylbenz-aldehyde, 3-methylbenzaldehyde, 4-methylbenzaldehyde, 2,3-dimethylbenzaldehyde, 2,4-dimethylbenzaldehyde, 2,5-dimethylbenzaldehyde, and 2,4,5-trimethylbenzaldehyde were selected as the methylbenzaldehyde and hydroxybenzaldehyde analogues (Fig. 1). For the vapor action against *T*. *putrescentiae* (Table 4), 2,4,5-trimethylbenzaldehyde (LD_50_, 0.78 μg/cm^3^) was about 20.18 times more toxic than benzyl benzoate (15.74 μg/cm^3^), followed by 2,4-methylbenzaldehyde (1.14 μg/cm^3^), 2,5-dimethylbenzaldehyde (1.29 μg/cm^3^), 2-methylbenzaldehyde (1.32 μg/cm^3^), 2,3-dimethylbenzaldehyde (1.55 μg/cm^3^), 3-methylbenzaldehyde (1.97 μg/cm^3^), and 4-methylbenzaldehyde (2.34 μg/cm^3^). For the contact action (Table 5), 2,4,5-trimethylbenzaldehyde (LD_50_, 0.54 μg/cm^2^) was about 22.22 times more toxic than benzyl benzoate (LD_50_, 12.0 μg/cm^2^), followed by 2-dimethylbenzaldehyde (0.89 μg/cm^2^), 2,3-dimethylbenzaldehyde (1.02 μg/cm^2^), 2,5-dimethylbenzaldehyde (1.11 μg/cm^2^), 2,4-dimethylbenzaldehyde (1.17 μg/cm^2^), 3-methylbenzaldehyde (1.38 μg/cm^2^), and 4-methylbenzaldehyde (1.78 μg/cm^2^). However, failed to show a acaricidal effect of the vapor (>19.5 μg/cm^3^) and contact actions (>13.0 μg/cm^2^) of 4-hydroxybenzaldehyde, 3-hydroxybenzaldehyde, 2-hydroxybenzaldehyde, 2,5-dihydroxybenzaldehyde, 2,4-dihydroxybenzaldehyde, 2,3-dihydroxybenzaldehyde, 3,4,5-trihydroxybenzaldehyde, 2,4,5-trihydroxybenzaldehyde, and 2,3,4-trihydroxybenzaldehyde at the highest concentrations tested against *T*. *putrescentiae*. In this regard, the methylbenzaldehyde analogues were more toxic than hydrobenzaldehyde and benzyl benzoate against *T*. *putrescentiae*, as has been described by some studies^28, 29^. Oh *et al*.^28^ reported that the methylaceotphenone analogues (2′-, 3′-, and 4′-methylacetophenone) possessed potent toxicity against *T*. *putrescentiae*, *Dermatophagoides pteronyssinus*, and *D*. *farinae*, but the hydroxyacetophenone analogues (2′,4′- and 2′,6′-dihydroxyacetophenone) had no acaricidal toxicity. Furthermore, Lee & Lee^29^ suggested that the acaricidal toxicity of 2-methyl-1,4-naphthoquinone containing the CH_3_ functional group on 1,4-naphthoquinone was greater than that of 2-hydroxy-1,4-naphthoquinone conjugating the OH functional group on 1,4-naphthoquinone against *T*. *putrescentiae*, *D*. *pteronyssinus*, and *D*. *farinae*. The lack of the acaricidal activity of hydroxybenzaldehyde analogues is may be connected to lack of CH_3_ functional group. In this regard, 2,4,5-trimethylbenzaldehyde which is conjugated with three CH_3_ functional group at position 2′-, 4′-, and 5′, exhibited the highest vapor and contact toxicities against *T*. *putrescentiae*.

**Table 4:** Acaricidal toxicity of hydroxybenzaldehyde analogues, methylbenzaldehyde analogues and synthetic acaricide against *T*. *putrescentiae*, using a vapor bioassay (^a^LD_50_/LD_95_ is the average of 5 determinations, with 30 adult mites per replication.

Compounds	LD_50_ (95% CL) (μg/cm^3^)^a^	LD_95_ (95% CI) (μg/cm^3^)^a^	Slope	χ^2^ value (df, *p*)	RT_50_^b^
3-Hydroxybenzaldehyde	>19.50	>19.50	—	—	—
4-Hydroxybenzaldehyde	>19.50	>19.50	—	—	—
2,3-Dihydroxybenzaldehyde	>19.50	>19.50	—	—	—
2,4-Dihydroxybenzaldehyde	>19.50	>19.50	—	—	—
2,5-Dihydroxybenzaldehyde	>19.50	>19.50	—	—	—
2,3,4-Trihydroxybenzladehyde	>19.50	>19.50	—	—	—
2,4,5-Trihydroxybenzladehyde	>19.50	>19.50	—	—	—
3,4,5-Trihydroxybenzladehyde	>19.50	>19.50	—	—	—
2-Methylbenzaldehyde	1.32 (0.99–1.62)	4.69 (3.54–6.67)	2.96 ± 0.42	2.448 (5, 0.784)	11.92
3-Methylbenzaldehyde	1.97 (1.54–2.38)	7.59 (6.23–10.85)	2.79 ± 0.38	8.034 (6, 0.236)	7.99
4-Methylbenzaldehyde	2.34 (1.91–2.82)	7.68 (5.27–11.18)	2.62 ± 0.40	8.086 (5, 0.152)	6.73
2,3-Dimethylbenzaldehyde	1.55 (1.19–2.07)	5.78 (4.18–8.82)	2.58 ± 0.38	4.690 (5, 0.455)	10.15
2,4-Dimethylbenzaldehyde	1.14 (0.87–1.48)	4.76 (3.78–7.06)	2.71 ± 0.40	4.857 (5, 0.434)	13.81
2,5-Dimethylbenzaldehyde	1.29 (0.91–1.52)	6.10 (4.67–8.88)	2.40 ± 0.37	7.265 (5, 0.202)	12.20
2,4,5-Trimethylbenzaldehyde	0.78 (0.55–0.92)	2.73 (2.11–3.94)	2.96 ± 0.53	3.533 (4, 0.473)	20.18
Benzyl benzoate	15.74 (13.81–17.76)	38.68 (32.14–48.84)	4.53 ± 0.64	2.492 (4, 0.646)	1.00
Negative control	>19.50	>19.50	—	—	—

^b^RT_50_, Relative toxicity = LD_50_ value of benzyl benzoate/LD_50_ value of each compound; Exposed for 24 h).

**Table 5:** Acaricidal toxicity of hydroxybenzaldehyde analogues, methylbenzaldehyde analogues and synthetic acaricide against *T*. *putrescentiae*, using a contact bioassay (^a^LD_50_/LD_95_ is the average of 5 determinations, with 30 adult mites per replication.

Compounds	LD_50_ (95% CL) (μg/cm^2^)	LD_95_ (95% CL) (μg/cm^2^)	Slope	χ^2^ value (df, *p*)	RT_50_^b^
3-Hydroxybenzaldehyde	>13.0	>13.0	—	—	—
4-Hydroxybenzaldehyde	>13.0	>13.0	—	—	—
2,3-Dihydroxybenzaldehyde	>13.0	>13.0	—	—	—
2,4-Dihydroxybenzaldehyde	>13.0	>13.0	—	—	—
2,5-Dihydroxybenzaldehyde	>13.0	>13.0	—	—	—
2,3,4-Trihydroxybenzladehyde	>13.0	>13.0	—	—	—
2,4,5-Trihydroxybenzladehyde	>13.0	>13.0	—	—	—
3,4,5-Trihydroxybenzladehyde	>13.0	>13.0	—	—	—
2-Methylbenzaldehyde	0.89 (0.69–1.11)	4.23 (3.16–6.40)	2.18 ± 0.29	7.327 (5, 0.197)	13.48
3-Methylbenzaldehyde	1.38 (1.03–1.74)	6.26 (4.80–9.33)	2.21 ± 0.28	5.518 (4, 0.238)	8.70
4-Methylbenzaldehyde	1.78 (1.39–2.14)	7.68 (5.82–10.85)	2.51 ± 0.30	3.470 (4, 0.482)	6.74
2,3-Dimethylbenzaldehyde	1.02 (0.84–1.26)	2.74 (2.17–4.11)	3.89 ± 0.60	2.462 (4, 0.651)	11.76
2,4-Dimethylbenzaldehyde	1.17 (0.98–1.38)	3.57 (2.84–4.61)	3.39 ± 0.41	3.318 (5, 0.651)	10.26
2,5-Dimethylbenzaldehyde	1.11 (0.86–1.37)	4.52 (2.84–5.98)	2.97 ± 0.39	1.542 (4, 0.819)	10.81
2,4,5-Trimethylbenzaldehyde	0.54 (0.43–0.71)	3.26 (2.40–5.48)	2.01 ± 0.30	3.498 (4, 0.478)	22.22
Benzyl benzoate	12.0 (10.56–14.01)	32.38 (25.66–43.29)	3.23 ± 0.44	2.645 (4, 0.619)	1.00
Negative control	>13.0	>13.0	—	—	—

^b^RT_50_, Relative toxicity = LD_50_ value of benzyl benzoate/LD_50_ value of each compound; Exposed for 24 h).

**Figure 2:**
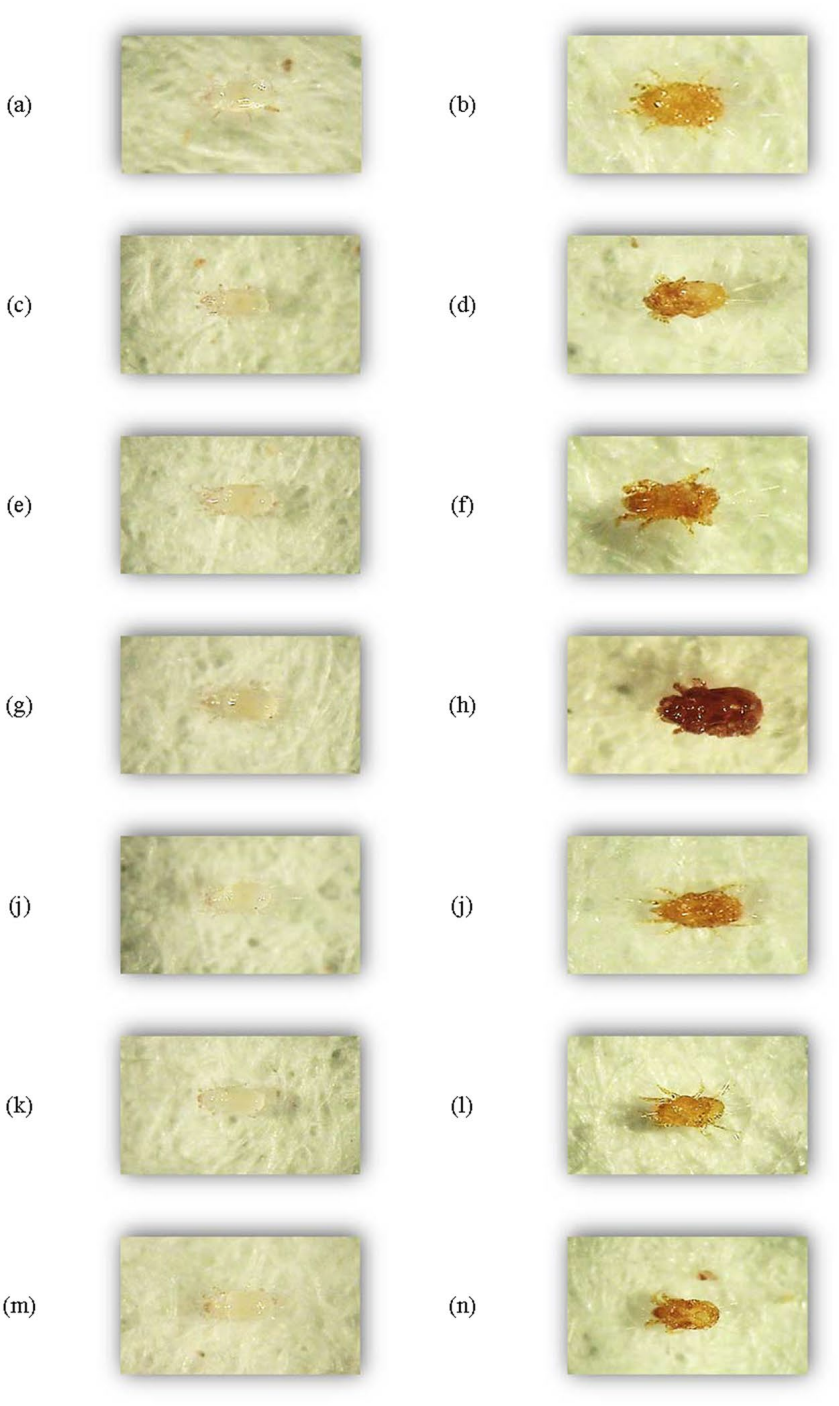
Color deformation of hydroxybenzaldehyde and methylbenzaldehyde analogues with (mixed at 9:1 ratio) and without 2,3-dihydroxybenzaldehyde to *T*. *putrescentiae* for 24 h at a dose of each LD_95_ values. (**a**) 2-Methylbenzaldehyde without 2,3-dihydroxybenzaldehyde; (**b**) 2-Methylbenzaldehyde with 2,3-dihydroxybenzaldehyde; (**c**) 3-Methylbenzaldehyde without 2,3-dihydroxybenzaldehyde; (**d**) 3-Methylbenzaldehyde with 2,3-dihydroxybenzaldehyde; (**e**) 4-Methylbenzaldehyde without 2,3-dihydroxybenzaldehyde; (**f**) 4-Methylbenzaldehyde with 2,3-dihydroxybenzaldehyde; (**g**) 2,3-Dimethylbenzaldehyde without 2,3-dihydroxybenzaldehyde; (**h**) 2,3-Dimethylbenzaldehyde with 2,3-dihydroxybenzaldehyde; (**i**) 2,4-Dimethylbenzaldehyde without 2,3-dihydroxybenzaldehyde; (**j**) 2,4-Dimethylbenzaldehyde with 2,3-dihydroxybenzaldehyde; (**k**) 2,5-Dimethylbenzaldehyde without 2,3-dihydroxybenzaldehyde; (**l**) 2,5-Dimethylbenzaldehyde with 2,3-dihydroxybenzaldehyde; (**m**) 2,4,5-Trimethylbenzaldehyde without 2,3-dihydroxybenzaldehyde; (**n**) 2,4,5-Trimethylbenzaldehyde with 2,3-dihydroxybenzaldehyde.

The color deformation of *T*. *putrescentiae* when treated and not treated with the methylbenzaldehyde and hydroxybenzaldehyde analogues was viewed with a microscope. Specifically, the cuticle of *T*. *putrescentiae* treated with 2,3-dihydroxybenzaldehyde showed color deformation to reddish brown, and stored food mites not treated with 2,3-dihydroxybenzaldehyde were colorless (see Supplementary Fig. S1). The color deformation of *T*. *putrescentiae* by the other methylbenzaldehyde and hydroxybenzaldehyde analogues was not observed in the vapor and contact actions, with the exception of 2,3-dihydroxybenzaldehyde. Therefore, we performed more in-depth tests of color deformation effects in order to determine 2,3-dihydroxybenzaldehyde to use as acaricidal additive of color deformation against *T*. *putrescentiae* (Fig. 2). The color deformation of seven methylbenzaldehyde analogues mixed with 2,3-dihydroxybenzaldehyde, respectively at 9:1 to 1:9 ratio against *T*. *putrescentiae* showed color deformation to reddish brown and valuable to distinguish with the naked eye. There was no significant difference in color deformation effects among ten ratios of each compound (9:1 to 1:9). According to a previous study, the color deformation of the insect and mite cuticles is related to benzene metabolism by the defense system in plants and action of phenoloxidase^30, 31^. Phenoloxidase is uniquely related to physiologically important biochemical processes, such as sclerotization of cuticle, defensive encapsulation, and melanization of foreign organisms^30^. Xue^31^ reported that the hydroxybenzaldehyde analogue, 2-hydroxybenzaldehyde, exhibited inhibitory effects on phenoloxidase against *Pieris rapae* larvae. Several researches have argued that high levels of cuticular dopamine affect the black pigment melanin in *Blattella germanica* ^32^, *Drosophila melanogaster* ^33^, *Manduca sexta* ^34^, and *Tribolium castaneum* ^35^. In addition, according to a previous study, the synthesis of *N*-β-alanyldopamine allows for sclerotization of proteins and brown cuticle pigmentation^34, 35^.

Based on the Wadley’s determination for the vapor action (Table 6), 2-methylbenzaldehyde (R_50_ = 0.98, R_95_ = 0.73), 3-methylbenzaldehyde (R_50_ = 1.19, R_95_ = 1.03), 4-methylbenzaldehyde (R_50_ = 1.38, R_95_ = 1.36), 2,3-dimethylbenzaldehyde (R_50_ = 0.98, R_95_ = 0.94), 2,4-imethylbenzaldehyde (R_50_ = 0.76, R_95_ = 0.93), 2,5-dimethylbenzaldehyde (R_50_ = 1.17, R_95_ = 1.24), and 2,4,5-dimethylbenzaldehyde (R_50_ = 1.02, R_95_ = 0.92) mixed with 2,3-dihydroxybenzaldehyde respectively, showed additive interactions. For the contact action (Table 7), 2-methylbenzaldehyde (R_50_ = 1.18, R_95_ = 0.97), 3-methylbenzaldehyde (R_50_ = 1.30, R_95_ = 1.35), 4-methylbenzaldehyde (R_50_ = 1.64, R_95_ = 1.57), 2,3-dimethylbenzaldehyde (R_50_ = 0.93, R_95_ = 0.60), 2,4-imethylbenzaldehyde (R_50_ = 1.04, R_95_ = 0.95), and 2,4,5-dimethylbenzaldehyde (R_50_ = 0.97, R_95_ = 1.48) mixed with 2,3-dihydroxybenzaldehyde respectively, showed additive relationship. When 4-methylbenzaldehyde or 2,5-dimethylbenzaldehyde were mixed with 2,3-dihydroxybenzaldehyde, respectively, 4-methylbenzaldehyde +2,3-dihydroxybenzaldehyde (R_50_ = 1.64, R_95_ = 1.57) and 2,5-dimethylbenzaldehyde +2,3-dihydroxybenzaldehyde (R_50_ = 1.63, R_95_ = 1.60) showed synergistic interactions. These findings demonstrate that 2,3-dihydroxybenzaldehyde changed the color of *T*. *putrescentiae* from colorless to reddish brown, but does not affect the acaricidal activities of methylbenzaldehyde against *T*. *putrescentiae*. Synergistic and antagonistic acaricidal and insecticidal effects have been observed in between essential oils as well as between components of essential oils^36, 37^. Previous study found that synergistic insecticidal interaction between camphor and 1,8-cineol to *Trichoplusia ni* is connected with the enhanced penetration of camphor^38^. In our study, no antagonistic interaction was observed indicating that the color deformation effects of 2,3-dihydroxybenzaldehyde on *T*. *putrescentiae* was independent of acaricidal activity of the methylbenzaldehyde analogues.

**Table 6:** Comparative acaricidal activity by vapor bioassays of benzaldehyde analogues with 2,3-dihyderoxybenzaldehyde against *T*. *putrescentiae* (^a^Each chemical mixed at 9:1 ratio with 2,3-dihydroxybenzaldehyde.

Chemical	Each chemical with 2,3-dihydroxybenzaldehyde^a^									
	Observed values (μg/cm^3^)^b^				Expected values (μg/cm^3^)^b^					
	LD_50_ (95% CL)	LD_95_ (95% CL)	Slope	χ^2^ value	LD_50_ (Wadley)^c^	LD_95_ (Wadley)^c^	R_50_^d^	R_95_^d^	S_50_^e^	S_95_^e^
2-Methylbenzaldehyde	1.48 (1.12–1.90)	6.94 (5.17–9.01)	2.58 ± 0.38	4.275 (5, 0.511)	1.45	5.07	0.98	0.73	Add	Add
3-Methylbenzaldehyde	1.81 (1.42–2.25)	7.83 (6.20–11.52)	2.60 ± 0.36	9.079 (6, 0.169)	2.16	8.08	1.19	1.03	Add	Add
4-Methylbenzaldehyde	1.85 (1.52–2.28)	6.01 (4.62–9.14)	3.25 ± 0.45	2.480 (5, 0.779)	2.56	8.17	1.38	1.36	Add	Add
2,3-Dimethylbenzaldehyde	1.74 (1.37–2.13)	6.60 (5.18–9.11)	2.69 ± 0.39	4.671 (5, 0.457)	1.71	6.21	0.98	0.94	Add	Add
2,4-Dimethylbenzaldehyde	1.66 (1.28–1.98)	5.55 (4.21–7.85)	3.09 ± 0.43	6.376 (5, 0.271)	1.26	5.15	0.76	0.93	Add	Add
2,5-Dimethylbenzaldehyde	1.21 (0.96–1.54)	5.28 (4.04–7.96)	2.66 ± 0.43	3.785 (4, 0.436)	1.42	6.55	1.17	1.24	Add	Add
2,4,5-Trimethylbenzaldehyde	0.84 (0.56–1.11)	3.24 (2.14–4.77)	2.40 ± 0.39	6.401 (6, 0.380)	0.86	2.99	1.02	0.92	Add	Add

^b^Expected LD_50_ based on Wadley’s calculation model. ^c^Wadley’s calculation of expected LD_50_ and LD_95_. ^d^Synergy ratio from Wadley’s calculation of expected LD_50_ and LD_95_. ^e^Determination of interaction of the mixture based on Wadley’s determination method: when R > 1.5, synergistic (Syn) interaction; when 1.5 ≥ R > 0.5, additive (Add) interaction; when R ≤ 0.5, antagonistic interaction).

**Table 7:** Comparative acaricidal activity by contact bioassays of benzaldehyde analogues with 2,3-dihyderoxybenzaldehyde against *T*. *putrescentiae* (^a^Each chemical mixed at 9:1 ratio with 2,3-dihydroxybenzaldehyde.

Chemical	Each chemical with 2,3-dihydroxybenzaldehyde^a^									
	Observed values (μg/cm^2^)				Expected values (μg/cm^2^)^b^					
	LD_50_ (95% CL)	LD_95_ (95% CL)	Slope	χ^2^ value	LD_50_ (Wadley)^c^	LD_95_ (Wadley)^c^	R_50_^d^	R_95_^d^	S_50_^e^	S_95_^e^
2-Methylbenzaldehyde	0.83 (0.66–0.1.01)	4.66 (2.39–5.18)	2.16 ± 0.31	3.511 (4, 0.476)	0.98	4.54	1.18	0.97	Add	Add
3-Methylbenzaldehyde	1.16 (0.97–1.37)	4.89 (3.34–6.78)	1.96 ± 0.24	3.539 (4, 0.472)	1.51	6.60	1.30	1.35	Add	Add
4-Methylbenzaldehyde	1.19 (0.99–1.41)	5.11 (3.89–8.43)	2.01 ± 0.27	4.011 (5, 0.404)	1.95	8.01	1.64	1.57	Syn	Syn
2,3-Dimethylbenzaldehyde	1.21 (1.03–1.54)	4.91 (3.67–6.89)	2.88 ± 0.36	3.298 (4, 0.509)	1.12	2.97	0.93	0.60	Add	Add
2,4-Dimethylbenzaldehyde	1.24 (1.01–1.46)	4.06 (3.21–5.47)	3.27 ± 0.40	4.953 (5, 0.422)	1.29	3.85	1.04	0.95	Add	Add
2,5-Dimethylbenzaldehyde	0.75 (0.55–0.88)	3.03 (2.36–4.57)	2.79 ± 0.39	4.347 (5, 0.501)	1.22	4.84	1.63	1.60	Syn	Syn
2,4,5-Trimethylbenzaldehyde	0.61 (0.48–0.84)	4.33 (3.18–6.28)	2.85 ± 0.44	4.126 (5, 0.531)	0.59	3.52	0.97	1.48	Add	Add

^b^Expected LD_50_ based on Wadley’s calculation model. ^c^Wadley’s calculation of expected LD_50_ and LD_95_. ^d^Synergy ratio from Wadley’s calculation of expected LD_50_ and LD_95_. ^e^Determination of interaction of the mixture based on Wadley’s determination method: when R > 1.5, synergistic (Syn) interaction; when 1.5 ≥ R > 0.5, additive (Add) interaction; when R ≤ 0.5, antagonistic interaction).

The present results implicate *M*. *arvensis* oil, 3-methylbenzaldehyde and its structurally related analogues as promising natural products of acaricides against *T*. *putrescentiae*. Interestingly, color deformation on the cuticle of *T*. *putrescentiae* from transparent to reddish brown was observed with the treatment of methylbenzaldehyde analogues with 2,3-dihydroxybenzaldehyde. In this regard, 2,3-dihydroxybenzaldehyde could be use as the acaricide additive for color deformation to protect from fall in the commercial value of stored food products. Since most mites are invisible to the naked eye, infestations can be difficult to detect until the mites become problematic. A major benefit of this methods is that it can be detected by changing the color of the *T*. *putrescentiae*. In the registration process, the fact that *M*. *arvensis* is inexpensive plant which can be easily cultivated, the cost would not be a problem for the commercial development of 3-methylbenzaldehyde isolated from *M*. *arvensis*. As to questions on possible toxicity of *M*. *arvensis* oil, the fact that it is treatment of malignant tumor of the oral cavity as folk medicine is indicative of its non-toxicity to humans^19^. Factors such as compound cost, dose, persistence, volatility and availability will be important, but determining the effective method for field application of acaricide will be crucial to success. Methods for combining our compounds with diatomaceous earth (DE) may be appropriate and adaptable for field application in organic farming. There is strong evidence that DEs can be successfully used in combination with other control strategies such as entomopathogenic fungi^38^, plant derivatives^39^, low doses of pyrethroids^40^, or even predators^41^. Therefore, further investigation of the interaction between mite kit and other acaricide, is necessary to exploit this promise.

## Materials and Methods

### Chemicals and sample preparation

Benzyl benzoate (99%), butyl isothiocyanate (98%), 3-chloro-2,4-pentanedione (95%), diacetone alcohol (98%), 2,3-dihydroxybenzaldehyde (98%), 2,4-dihydroxybenzaldehyde (97%), 2,5-dihydroxybenzaldehyde (98%), 2,3-dimethylbenzaldehyde (97%), 2,4-dimethylbenzaldehyde (90%), 2,5-dimethylbenzaldehyde (99%), dodecane (99%), hexachloroethane (99%), hexadecanoic acid (99%), 3-hydroxybenzaldehyde (99%), 4-hydroxybenzaldehyde (98%), 2-methylbenzaldehyde (97%), 3-methylbenzaldehyde (97%), 4-methylbenzaldehyde (97%), nonanal (95%), octanal (99%), 3-octanone (98%), oleamide (99%), 2-pentylfuran (98%), phenylacetaldehyde (90%), 2-phenyl-2-imidazoline (98%), pentadecane (99%), tetradecanoic acid (99%), 1,1,3,5-tetramethylcyclohexane (95%), 1-tridecanol (97%), 1,2,3-trimethylbenzene (90%), 2,4,5-trihydroxybenzaldehyde (99%), 2,4,5-trimethylbenzaldehyde (97%), 3,4,5-trihydroxybenzaldehyde (98%), and 2,3,4-trihydroxybenzaldehyde (98%) were obtained from Tokyo Chemical Industry (Tokyo, Japan) and Sigma (St. Louis, MO, USA). The *Myosotis arvensis* L. aerial parts (50 g) and seeds roots (50 g) were purchased from an herbal store and extracted by steam distillation^22^. Essential oils were concentrated using an evaporator at 26 °C.

### Rearing of *T. putrescentiae*

The rearing method for *T*. *putrescentiae* modified by Yang *et al*.^42^ was utilized. Food and grain feed was made up of yeast and fry powder located in the rearing plastic box (16 × 12 × 5.9 cm) at 24.9 °C and 74.6% relative humidity in an incubator. Protein content in the powder was over 48.9%.

### Acaricidal toxicity

The acaricidal toxicities of 3-methylbenzaldehyde derived from *M*. *arvensis* oil and its analogues were measured with the contact and vapor methods against *T*. *putrescentiae*. The contact and vapor methods were slightly modified from the method described by Lee and Lee^43^. The sample concentrations were a wide range from 20.0–0.02 μg/cm^2^. The sample dissolved in acetone (10 μL) were applied to filter paper (1 mm thickness × 8 mm i.d.), and dried for 11 min. The filter paper was moisturized by 5 μL distilled water and then placed in the cap of a microtube (2 mL, Greiner Bio-One GmbH, Germany). After preparing the bioassay, groups consisting of 30 randomly selected adult mites (7–10 days old) were inoculated in each microtubes, and the lid was closed. Acetone and benzyl benzoate was applied as the negative control and the positive control, respectively. For the vapor action, various concentration (20.0–0.02 μg/cm^3^) of test samples were applied to the filter paper (55 μm thickness × 5 cm). Each filter paper was placed in the petri dish (8 mm deep × 5 cm i.d.) lid after the treated and dried for 11 min. The filter paper was moisturized by 20 μL distilled water and then mites of 30 individuals (7–10 days old) were separately inoculated in each petri dish. Treatments for the contact and vapor methods were repeated five times in an incubator for 24 h at 27 ± 1 °C and 75% relative humidity in darkness. Dead mites were confirmed under a microscope (×20).

### GC-MS

The constituents of the essential oil derived from *M*. *arvensis* leaves were measured with the Hewlett-Packard HP 6890 and H5973 IV series (Agilent, USA) and were separated with HP−Innowax capillary column and DB−5 column (0.25 μm thickness × 2,990 cm L. × 0.25 mm i.d.). The conditions of the GC column were as follows: helium at 0.75 mL/min; column temperature (50 to 201 °C) at 2 °C/min; injector temperature (211 °C); split ration (48:1); ion source temperature (231 °C); ionization potential (70 eV); mass spectra range, 50–800 amu. The constituents of *M*. *arvensis* oils were evaluated according to the retention times, retention indices, and mass spectra and were identified by comparison with a spectrum library. The relative compositions of *M*. *arvensis* oil constituents (%) were measured by comparison with internal standards.

### Color deformation effects of methylbenzaldehyde analogues with acaricidal additive against *T*. *putrescentiae*

To evaluate color deformation effects of the methylbenzaldehyde analogues (4-methylbenzaldehyde, 3-methylbenzaldehyde, 2-methylbenzaldehyde, 2,5-dimethylbenzaldehyde, 2,4-dimethylbenzaldehyde, 2,3-dimethylbenzaldehyde, and 2,4,5-trimethylbenzaldehyde) with color deformation kit, 2,3-dihydroxybenzaldehyde, mixtures were prepared a 9:1 to 1:9 ratio of the compounds. Mixtures were applied to *T*. *putrescentiae* by contact bioassay at dose of LD_95_ value and their color of cuticle was estimated using an optical microscope.

### Structural relationships between methylbenzaldehyde analogues and 2,3-dihydroxyben-zaldehyde as acaricidal additive for color deformation against *T*. *putrescentiae*

To evaluate structural relationship between methylbenzaldehyde analogues and 2,3-dihydroxybenzaldehyde against *T*. *putrescentiae*, the mixture was prepared a 9:1 ratio of the compounds based on color deformation effect. The acaricidal toxicities of the mixture was measured with the contact and vapor methods against *T*. *putrescentiae*. To determine the structural relationships between the methylbenzaldehyde analogues and 2,3-dihydroxybenzaldehyde, we used statistical model to compare expected and observed LD_50_ and LD_95_ values: Wadley’s model^37, 44^. The interaction between the expected and observed LD_50_ and LD_95_ values were compared as R = expected LD_50_ (LD_95_)/observed LD_50_ (LD_95_). The relationship between the methylbenzaldehyde analogues and 2,3-dihydroxybenzaldehyde as defined as either synergistic (when R > 1.5), additive (1.5 ≥ R > 0.5) or antagonistic (R ≤ 0.5) based on above model^37^.

### Statistics

Data obtained for each dose response bioassay were subjected to probit analysis. The LD_50_ value, LD_95_ value and the slope of the regression lines were determined by statistical package SPSS, version 12.0 for Windows. Relative toxicity (RT) was determined by the ratio of the commercial acaricide LD_50_ value to the LD_50_ value observed for each compound^45^.

## Electronic supplementary material

Supplementary Information
