# Supplementary material for: Food Protective Effects of 3-Methylbenzaldehyde Derived from Myosotis arvensis and Its Analogues against Tyrophagus putrescentiae
Source: Sci Rep. 2017 Jul 26;7:6608. doi: 10.1038/s41598-017-07001-5 (PMC5529470; doi:10.1038/s41598-017-07001-5)

Food Protective Effects of 3-Methylbenzaldehyde Derived from *Myosotis arvensis* and Its Analogues against *Tyrophagus putrescentiae*.

Jun−Hwan Park[1], Na-Hyun Lee[2], Young-Cheol Yang[1] & Hoi-Seon Lee[1]

[1]Department of Bioenvironmental Chemistry, Chonbuk National University, Jeonju 54896, Korea

[2]School of Chemical Engineering, Chonbuk National University, Jeonju 54896, Korea

Supplementary Figure S1. Food mite kit against *T. putrescentiae*. (A) food mites, not treated (× 100), (B) food mites, treated with 2,3-dihydroxybenzaldehyde (× 100), (C) food mites, treated with 2,3−dihydroxybenzaldehyde (actual size).

(A)

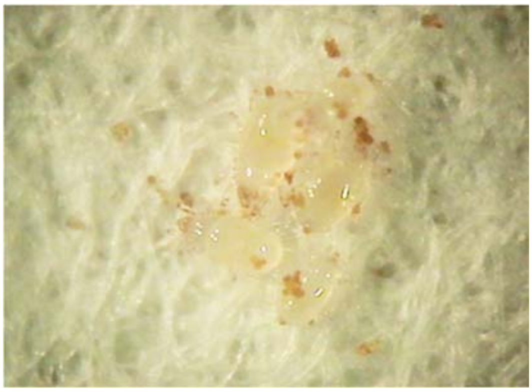

(B)

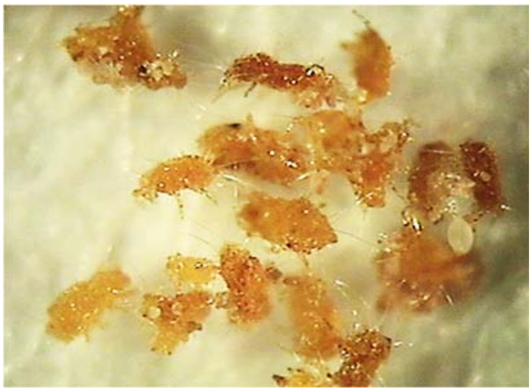

(C)

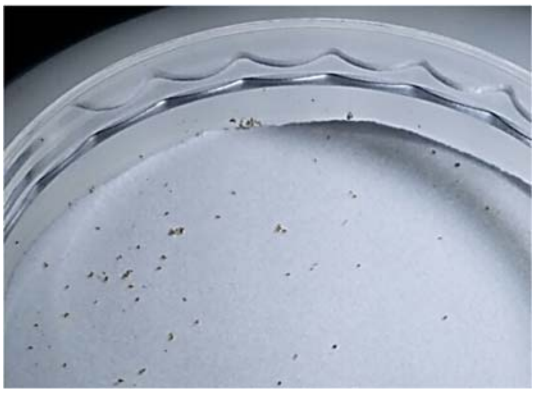

Supplement: Supplementary file 1 — Supplementary Information [file 41598_2017_7001_MOESM1_ESM.pdf]
